# The Fourth Annual Report of the Registrar-General of Births, Deaths and Marriages in England

**Published:** 1843-10-01

**Authors:** 


### The Fourth Annual Report of the Registrar-General of Births, Deaths and Marriages in England. 8vo. London, 1842. Pp. 361.

The reports of Births, Deaths and Marriages have never failed to convey much information, capable of the most useful application, and the more valuable because, until the last few years, a lamentable deficiency has been felt in our knowledge on the subjects of which these reports treat.

It is not surprising, therefore, that these volumes as they successively appear should present many imperfections—that, notwithstanding the large body of facts produced, no inquiry respecting the progress of the population and the causes influencing health and mortality can yet be carried out in a full, complete and satisfactory manner to its legitimate conclusion.

Of this fact the Registrar-General himself seems fully conscious—nor is it indeed possible that those who have devoted so much labour and industry to analyse and classify all the evidence furnished, could fail to be painfully convinced that we are yet unprovided with the statistical information which would render any deductions from them conclusive. The more deeply the subject is entered into the more clearly must discrepancies and imperfections appear; and, while new means are thus suggested of supplying in future years such deficiencies, the conviction is forced upon us, that more time is yet required to attain such results, and that at present we must receive any conclusions drawn, as approximations to the truth, rather than the verified products of an inquiry based upon figures and which therefore should yield the most accurate results.

We hail the appearance of this 4th Report, however, with pleasure, for whatever deficiencies yet remain, there is evidence that a steady advance is making towards the attainment of a more perfect system of registration, and that the attention of the Registrar-General is pointedly directed to the best means of removing all difficulties. Nor have the past exertions been without good fruit—as will be shown by reference to the details presented in this volume, which we have no hesitation in saying is a great improvement on the preceding. It comprises the Report of the Registrar-General, and the returns on which it is founded, showing the progress of population, the births, deaths, and marriages in four years—the increase and decrease of mortality—upon the whole and in different parts of the country and different cities—the mortality at different ages, &c.

To this is added an appendix, containing three letters or reports of Mr. Farr, to whose statistical labours the public are already indebted for many very valuable papers. The three reports refer to—

1. The Progress of Population—Births, Deaths, and Marriages—Fecundity of Females, &c., in which an attempt is made to deduce from facts some of the general laws by which they are regulated.
2. The Registration and Classification of Diseases as Causes of Death.
3. State of Public Health in 1840.

The bulk of the volume consists of numerous returns and tables, forming the annual abstracts upon which the report of the Registrar-General and the letter of Mr. Farr are founded—of which the causes of death throughout the statistical districts form a large proportion.

With this general outline of the contents and pretensions of the volume we proceed to give such an analysis of the leading facts as may lead to a correct appreciation of all the importance of the subject. However dry and uninteresting the numerical details may be from which the conclusions are drawn, the latter are pregnant with interest to the public generally, by their important influence and bearing on our social institutions and the well-being and prosperity of the mass—and they are, if possible, still more deeply fraught with interest to the medical profession, in whose charge is the public health—to whose suggestions and exertions chiefly must any ameliorations be attributed, and through whom the most valuable statistical information can alone be obtained.

Major Graham's report commences with a statement giving a comparative view of the number of births, deaths and marriages which were registered in the four years ending June 30, 1841, and it is shown that, while there is an increase in the births and deaths in 1840-1, compared with 1839-40, there is a considerable diminution in the number of marriages.* The marriages registered have been nearly 1 in 127: the births 1 in 32: the deaths 1 in 45 of the population.

Referring to certain modifications and improvements lately introduced by a new subdivision of the country into districts and sub-districts, and in the formation of the abstracts, the important objects in view are ably defined.

* The marriages relative to the female population, were nearly 3 per cent. less than in the preceding year, and we are informed this decrease was observed in all the divisions of the kingdom except the South-Eastern counties; it was most obvious in Yorkshire and the North-Western division.

"I have thus been enabled to exhibit the ages at which the people died in all the large town districts, in the populous manufacturing and mining districts, and in many of the agricultural districts, which have only been united when the population was scattered, and when the annual births and deaths were so few in number as to be severally inapplicable to any useful calculations. Every person is naturally most interested in the district in which he resides; and the present form of publication will afford the scientific inquirer the means of framing tables of mortality,—of calculating the mean duration of life—in his own district, and comparing the results with those deduced from the facts registered in neighbouring districts or in the whole kingdom. The inhabitants will thus learn from authentic documents the relative sanatory state of their neighbourhood; the causes of disease will be discovered, and suggestions which may lead to numerous improvements will be pressed upon the attention of the resident proprietors and authorities. The friendly societies will obtain from the local tables the means of adjusting their premiums equitably to the prevailing rate of sickness and mortality, which may be a fourth or a half higher in some districts than in others, and will be found to differ among the same classes, so as to affect materially the money value of assurance and the stability and prosperity of the societies. Materials of thought and guides of action will thus be afforded, on questions affecting health, life, and death, to scientific and benevolent persons in all parts of the kingdom." 3.

*Progress of Population.*

We find the population of England enumerated in the year 1821 comprized 3,105,895 more persons than had been enumerated in 1801, whilst the increase of numbers in the 20 years from the 31st of May 1821, to the 6th of June 1841, was 3,930 416, leaving the army, navy, marines and registered seamen out of the account.

To obviate this source of error arising from the difficulty in this country of ascertaining the exact number of males that should be taken into account, it has been deemed more relevant to the object of inquiry, to compare the marriages and the births with the females living, than, as has been more usually done, with the entire population.

Thus the increase of females in the 10 years 1831—1841, being 14·17 per cent., or at the rate of 1·334 per cent. annually, the Registrar-General has thought it better to assume at present that the whole population of the kingdom has increased within the last 10 years at the same rate as the females enumerated, rather than "attempt to fix the exact rate of increase among males by making a series of suppositions relative to the disturbing effect of the army, navy, marines, registered seamen, emigration, and the possible omissions in the five censuses."

The female population has been taken therefore as the basis of nearly all the comparisons.

MARRIAGE.

*Proportion of Minors to Adults married.*—The Registrar-General observes that—

"As the age at which marriages take place, in connexion with the increase of population, is supposed to have a great influence upon the misery or happiness of the people, I wish I could have stated the ages of the persons who were married in every county; but the exact ages were only specified in a small number of districts; and the number of districts in which this was done last year was less than in any previous year. The following tables, however, compiled from the Registers of three years, will show the ages at which 40,874 persons were married." 9.

"More than half of the men (10,383), and half of the women (10,424), were married between the ages of 20 and 25; 2,711 women, and 537 men were married under the age of 20. The mean age of the men was 27·30 years, of the women 25·35 years." 9.

### BIRTHS.

In reference to births there is a lamentable deficiency of authentic data, as the following remark attests.

"All the births are not registered. The number omitted amounts to several thousands. I hope that the registrars, when they are made acquainted with this fact, will be induced to employ more vigilance in discovering the births that occur in their districts; but I am of opinion that the registration of births will not be complete until it is enacted by law that the father or mother, or some other qualified informant, shall give notice within a fixed period to the registrar of a birth having taken place." 11.

### *Proportion of Males to Females Born.*

"The births of 760,983 boys, and of 725,689 girls, were registered in the three last years; so that, in 100,000 births, 51,187 were boys, 48,813 girls; or the boys were born in the proportion of 10,486 to 10,000 girls. The excess of males born was greatest in the northern, least in the southern parts of England. The greatest number of births is registered in the winter and spring quarters; in those quarters, also, the proportion of males to females born is greatest. In the two last years, 105 boys have been born to 100 girls, or 21 boys to every 20 girls." 12.

Notwithstanding this excess it is found that the greater mortality of males reduces them nearly to an equality with females by the age of 20, and at the last census the males were to the females enumerated as 10,000 males to 10,465 females. Thus, by the silent operation of certain general laws, does the Creator ensure that equilibrium and relative proportion of the sexes essential to the well-being of the species, and these tables afford many examples "of the secret adjustments" which exist and of the laws which regulate all *increase and decrease* of social combinations.

### *Mortality.*

It appears that more deaths were registered in the year ending the 30th of June 1841 than in any preceding year—making due allowance for the increase of population, it would seem that the mortality of that year slightly exceeded the mortality of the preceding year, and was nearly 6 per cent above that of the year 1838-9. There are no means of knowing what the average mortality actually was before the present system of registration was introduced—according however to Mr. Edmond's estimates the annual mortality of females was 2·05 per cent. during the 18 years, 1813—30.

The relative annual mortality per cent., as shown by the mortality of females was 2·055 and 2·157, and 2·171 in the three years. More deaths were registered in the year ending the 30th of June 1841, than in any preceding year—the number registered 355,622, having exceeded the deaths in the three.

Although the aggregate increase of mortality, if we assume Mr. Edmond's estimate to be correct, may not be deemed very considerable—yet it *is an increase*, and we are entitled to look for progress in a contrary direction. With the advance of civilization, of medical science, and the sciences generally, who can doubt that, if this increased knowledge of the causes of mortality and the influences injurious to health—together with increased means for combating them were largely and faithfully applied for the welfare of the mass of the population, there would be an obvious and marked decrease instead of increase in the mortality.

Whence then this retrograde course?

We must look for the causes, doubtless, in those conditions which influence the health of the great mass of the labouring and pauper population. The exposures with which the public press has been teeming of the recklessness with which human life is deteriorated and perilled, whenever large bodies are collected together—whether for labour in our manufactories or mines—parochial relief in our poor houses—or for punishment in our gaols and prisons, and the frequent violation of every principle of hygiene and of enlightened policy or humanity, manifested by those in whose hands fortune has placed the control of the conditions under which the labouring population must toil for their bread, or suffer charity or punishment, significantly tell the tale to which these statistical results do but point the moral!

Doubtless legislation cannot altogether supply that kindly sympathy which is so much wanting between those who command and employ, and those who serve, and of which we believe there is less in this country than in any other in Europe—still there is much left for legislation to do.

Lord Ashley's humane and disinterested efforts, evinced by the Factory and Colliery Bills, are in the right direction. Legislation may at least prevent such atrocities as a whole manufacturing population bringing up a decrepid, yet precocious and vicious generation, by compelling their children of four years of age to labour eight, ten, and twelve hours a day—and often through the night—before they attain the age of puberty.

So, in reference to the diet and system of gaols, and that of poorhouses, which it is the unfortunate tendency of the present administrative theories to assimilate with prisons—there is sufficient knowledge widely spread in the medical profession throughout the length and breadth of this kingdom—not only to enlighten the Legislature as to the true principles of hygiene and the nature and bearing of all the influences tending to impair health and destroy life—but judiciously to carry out the application of those principles which a truly enlightened policy would dictate.

Unfortunately for all parties—for the mass of the people not less than for the members of the profession, it happens that professional knowledge and acquirements have been valued at an inverse ratio with the facility with which they can be obtained. The supply having been over-abundant, and disproportioned to the demand; the usual consequences of an excess of products, have been in operation, and medical acquirements have been depreciated and undervalued.

Thus it is—that although a board of guardians would shrink from the disgrace and absurdity of advertising for a baker, who would contract to supply bread for paupers at a certain loss upon each pauper's supplies, or even for a coffin-maker, to furnish the materials for a burial of the valueless remains at less than prime cost, they do not scruple—basing their demand on the exigencies of his position in their parish—to call upon a medical man to pay a visit at the houses of poor patients, at varying distances, and not only to give them the benefit of his skill and the cost of time, *but medicine,* at the price of 3¼d. a visit—which by no possibility can cover the prime cost of the drugs as purchased in the market! Nay, their cost, if he be a conscientious man, must often be ten times the amount fixed for his visit, and the benefit of his time, skill, and drugs! Is not this a scandal to a Christian country and a system of pillage and robbery under a very flimsy mask?

While such things are—while such practices are tolerated in this country by the Legislature, the Government and society at large—it must be that misery and mortality will go on hand-in-hand increasing—and though the humanity of the mass of medical men full often supplies, gratuitously and most conscientiously, that care and attention which their guardians with shortsighted niggardliness—(wasteful improvidence in the long run)—refuse to provide for the poor—the mortality will increase, and the alienation of the labouring classes from the superior ranks is not likely to diminish.

The Alverstoke board of guardians have become a notable type of the heartlessness and stolidity which drives the destitute to extremities. In reference to some of the more ordinary causes of mortality dependent on adventitious circumstances and localities, the following remarks, coming from the Registrar-General, are worthy of attention.

"It has been known for many years that the mortality is, as a general rule, higher in cities than in the country; and it has always been a matter of much interest to know the relative mortality of different cities. Since it has been proved that the mortality of cities may be greatly reduced, the question has acquired additional interest from the facility it affords for analysing the causes of insalubrity, demonstrating their existence, and suggesting the means by which the ravages of diseases may be effectually arrested."

"The town mortality generally increases with the density of the districts; which would not be the case if the same neglect of the means of sewerage and purification did not pervade the worst parts of all of them to nearly an equal extent. The influence of site, habits, diet, and employments on the duration of the lives of the inhabitants can only be successfully investigated on the spot; and I am happy to learn that this important duty has been undertaken in Leeds, Birmingham, and other places, by the Town Councils and medical societies." 15.

The greatest mortality has been in Chester and Lancaster, forming the North Western Division; the lowest number of deaths in Wilts, Dorset, Devon, Cornwall and Somerset—forming the South Western Division. The mortality in the first division being nearly half as high again as in the second.

It appears, from a table furnished, that the increased mortality already referred to, fell upon the young and aged, the mortality of persons between the ages of twenty and sixty years, having varied very little in the four years.

This might, of course, have naturally been anticipated: when deleterious influences are brought to bear upon large numbers of mankind, it must ever be those at the beginning and decline of life, who principally sink under the diseased states generated.

*Education versus Marriage.*

Some curious details are furnished by the marriage registers, taken as tests of the state of education. The test is made with reference to writing, and it is well observed that, the simplicity of this test is its great recommendation.

"The parties are neither asked whether they can write or read—nor formally requested to write, but sign the marriage registers with their name or their mark in attesting the marriage, and the tables show the proportion who signed with marks. The parties who marry are, on an average, about twenty-five years of age, so the test shows the state of education ten or twenty years ago, and the subsequent inducements to the retaining of the information and skill then required."

"It appears, from the average of three years, that 33 men in 100 and 49 women in 100 signed with marks. It is, therefore, probable that only 67 men, and 51 women in 100 can write their own names."

It is further shown that there are great differences in the state of education, in different counties. Thus it is calculated that 84 in 100 men can write in Cumberland, and only 48 in Bedfordshire.

The Tabular Abstracts of Births—Deaths—Marriages and Ages at Death of Males and Females—are given in great detail, and they seem compiled with care, and must afford valuable tables of reference under their different heads.

Mr. Farr's three papers—the first on a subject connected with these abstracts, the second, on the classifying of the causes of death, and the third, on the state of the public health in the year 1840, afford some further elucidations—particularly the second and third, well repaying attentive consideration.

The investigation of epidemic diseases mentioned in the appendix to the last report, is still deferred, we are told, in order to render the series of facts more extensive—as also a promised paper on the causes of death at different ages. From Mr. Farr's known industry and ability, we look forward with pleasure to the appearance of these contributions to medical and statistical science.

The first of Mr. Farr's papers commences with an Analysis of the Increase of Population in geometrical progression.

"*How the rate of Increase is accelerated and retarded?*

A population increases in regular geometrical progression when the births exceed the deaths, and the ratio of the births and of the deaths to the population remains constant. Thus in England every 100 persons living in 1801 had increased to 132 in 1821; and every 100 persons living in 1821 had increased to 132 in 1841; the 100 persons living in 1801 had, therefore, increased to 175 in 1841, and at the same rate will amount to 200 in the year 1850, 300

in the year 1879. The mean rate of increase was ·0141 annually; that was probably the excess of the births over the deaths. Grain, fruit, animals, also, increase in geometrical progression; but the increase of capital, at compound interest, is the most familiar example of this kind of progression, and may render it intelligible to the general reader." 134.

*Proportion of Persons who marry in England—Marriage Age—Fecundity of Females.*

It appears that 10 in 20 of the people of this country are born in wedlock—and, by the census returns, that the number of women who attained the mean age of 24·3 years in the middle of 1840 was about 143,830, of which it is calculated 113,361 married for the first time. The result therefore is that 79 women in 100 who attain the age of 24 are married, and that 21 in 100 are never married.

About 132,236 men were enumerated at the mean age of 25·5 years at which men are first married. So that of 100 men enumerated who attain the average age at which marriage is consummated 82 do marry and 18 do not. Making allowance, however, for soldiers, sailors, &c. not included, it seems that the proportion of men who marry is only 78, or 1 less than the proportion which was found for the female sex.

It is further stated the returns show that, by re-marriages, about 100 women marry 108 men, and 100 men 113 women.

"More women are married than men; but the women are married at an earlier age, when the number of them living is greater than the number of men living at the age when men marry: so that, at the respective ages of marriage, about 79 in 100 of each sex marry. Of 100 women married, 8 were widows; of 100 men, 12 were widowers."

"As the number of marriages, however, has increased for many years, and the expectation of life among women at the nuptial age is greater than that of men, it is probable that about 1 in 3 widowers and 1 in 4 widows re-marry.

"The fact, that one-fifth of the people of this country who attain the age of marriage never marry; and that the women, though capable of bearing children at 16, and certainly nubile at 17, do not marry until they attain a mean age of 24.3, the men until they are 25½, proves that prudence, or 'moral restraint,' in Mr. Malthus's sense of the term, is in practical operation in England to an extent which had not been conceived, and will perhaps scarcely be credited when stated in numbers." 136.

It has been elsewhere laid down as an axiom, that poverty is the state in which population most rapidly advances, and that marriages are more frequent, more early, and more prolific, among the poor than among the rich: and this is accounted for by the supposition, that a man of education looks to advancement in the world before he saddles himself with the expenses and cares of domestic duties, whereas, as a poor man cannot be in a worse condition, he looks, on the contrary, to the possession of a wife and helpmate as the only solace at his command.

If this be true, and the tables at present furnished do not enable any very accurate test to be applied, it must be inferred that of the one-fifth who never marry, by far the larger proportion are people of education seeking for advancement as the first necessity of their lives, and yet too insecure to give "hostages to fortune," according to Bacon's definition, in the shape of a wife and children.

The influence of these circumstances on the progressive increase of the population and on the fecundity of marriage is very curiously yet clearly brought out.

"The actual fecundity of the married women of this country may probably be expressed accurately enough, if a correction be made for the increase of marriages, and for the illegitimate children borne before and after marriage by women who marry, at 5 children to every woman married, and 4.5 children to every wedding. The 5 children replace the 2 parents, and those persons who from early death or from other circumstances bear no children.

"The number of women living and enumerated, June 1840, was, in round numbers, 1,630,000 aged 15-25; 1,272,000 aged 25-35; 900,000 aged 35-45; and these three ages, at which 3,802,000 women were living may be considered the ages of childbearing, the middle period being that in which the greater number of children are produced.

"The 3,735,000 women living in the 2 years, June, 1839-41, between the ages 15-45, gave birth to 562,346 children annually: 66 women produced 10 children every year: only 1 in 7 women (6.6) at the childbearing age gave birth to a child in the year. Children are occasionally borne at 15, or as late in life as 55; but if the mothers of the 562,346 children had all been aged 17-40, there would have been only 1 annual birth to 5 women living of that age. It has been calculated that, on an average, 2 years intervene between the birth of every child; or that of 2 women one has a child every year. After a correction has been made for unprolific women, the difference between 1 in 2, and 1 in 5 or 6, corroborates the previous result, and shows how much, notwithstanding the increase of population, the reproductive force is repressed by prudence.

"The population of this country may have increased, and may increase by an augmentation in the number of marriages and births; or, by a diminution in the number of deaths, and the consequent prolongation of life. The annual number of births may be increased in two ways: by an increase of the number of persons married, and by earlier marriages, which shorten the interval elapsing between successive generations. Thus 113,361 women were annually married (for the first time) in each of the two years ending June 30th, 1841, when 160,000 women attained the age of 20. If 10,000 be subtracted for sickness, infirmity, and incapacities of various kinds, 150,000 will remain who might have married, and thus have augmented the numbers married by one-third (32.7) per cent. The increase by birth, exclusive of illegitimate children, is about 3.4 per cent. annually; and if the marriages and births be increased one-third, or in the above ratio, the increase by birth will rise to 4.3 per cent., leaving, after subtracting the loss by death, (which shall be supposed to remain stationary at 2.2 per cent.) instead of 1.3, the present rate, 2.1 per cent. annually as the rate of increase, raised to its height by the greater number of married child-bearing women.

"I shall not discuss the litigated question, whether early marriages are more fruitful than late marriages; for, if even women who married at a mean age of 30 bore as many children as women married at 20, it will be immediately perceived that the annual number of births, and the rate of increase, will be widely different in the two sets of circumstances. It may be assumed that at the birth of their children the age of the mothers will be advanced equally in both cases six years, for instance, on an average—from the time of marriage; the mean age at the time the children are born will consequently be 36 years and 26 years. The interval from the birth of the mothers to the birth of the children will be 36 years and 26 years; and, according to the same law, the interval from the marriage of the mothers to the marriage of the children will be equally 36 years and 26 years. Now, in this case, altogether independently of the reduction by death in the 10 years, if the same number of women continue to marry, and if the expectation of life and the fecundity of the women remain unchanged, the births will be raised above or depressed below the present number, in the inverse ratio of 36 and 26 to 30. At present, the interval from generation to generation, from the birth of the parents to the birth of their children, may be 30 years; in the case of the early marriages, a generation would be reproduced every 26 years; of the late marriages, every 36 years; and, as by the hypothesis, the number born in *each generation* would be the same, the number born *in a given time* would differ in the ratio of the intervals which separated the generations." 138.

"I do not advance the preceding numerical statements as absolutely correct or definitive; and I hope to be able to resume the examination of these important subjects at a future time, when more extensive materials have accumulated and have been analyzed. None of these qualifications will, however, invalidate the general principles; and the facts prove, beyond all question, that the population of the country is susceptible of an immense expansion; that it is voluntarily repressed, and always has been repressed, to an extent which has not been clearly conceived or stated; and that the means in the hands of nature, and of society, for increasing and diminishing the population are simple, efficient, and quite compatible with our ideas of the benevolence of the divine government of the world.

"Writers upon population have, perhaps, exaggerated the influence of the increase of population on the strength and prosperity of states; but its importance is unquestionable, and it must always be interesting to understand the laws which regulate the death—the reproduction of individuals; and which, in the midst of the struggles of the antagonist forces of disease and death, the losses by war, want, vice, and error, ensure the perpetuity and life of nations." 139.

"When the rate of increase is to be lowered, the usual course appears to be to defer to the extent required the period of marriage. If the supplies of subsistence were cut off, if science and industry were unable to convert a larger proportion of the materials of nature into food, and all the outlets and demands of emigration were closed, the population might unquestionably be brought to a stationary condition without increasing the deaths—*by reducing the number of marriages.* At present one-fifth of the women who attain the age of 24.3 years never marry; if one-half of the women who attain that age never married, and illegitimate births did not increase, the births would ultimately not exceed the deaths, and the population would remain stationary. But the same end would be almost as effectually and less harshly attained, though four-fifths of the women who arrived at the mean age of marriage continued to marry, if instead of beginning to marry at 18, none married under 23, and the mean age of marriage were raised to 30 years; for the interval from generation to generation would be thus extended, the children to a marriage diminished, and the number of women at 30 would be reduced by the loss of the younger lives." 140.

And thus Mr. Farr would show that if any part of the population of this country is increasing too fast, "the means of repression are simple, would not be harsh in their operation, and are at the command of the immediate sufferers." Again, he adds—

"Should the time nevertheless come, when the country is sufficiently populous, and it should be desirable to retard or stop the progress of population—the analysis of the marriages, births, and deaths, in connexion with the census returns, will show, as has been already proved, that this may be effected without raising the mortality. The principle of 'an increase of the population in geometrical progression' has nothing in it fatal, irresistible, inexorable; upon a rigorous analysis of the facts, it is seen that it consists of nothing but an excess of births over the deaths, and becomes a negative quantity, or 'a decrease of population in geometrical progression,' if the births cease to maintain the same ratio to the population; and the births may always be reduced rapidly by retarding the period and number of marriages: so that the mathematical terror, 'a geometrical progression,' cannot alarm any one in the light of day. I do not desire to disguise or underrate the gravity of the fact, that the population of England has increased, as the censuses prove,—and the excess of births over deaths leaves beyond doubt—in a geometrical progression for 40 years, and at a rate by which, if continued, it will double every 49 years."

This very able paper concludes with the following consolatory remarks for the disciples of Malthus.

"Both these writers (Dr. Price and Mr. Malthus) contributed essentially to the development of the true theory of population; both rendered important services to mankind by their investigations; but the facts since elicited, and the further prosecution of the inquiries which they commenced, have shown that, while the study of the doctrine of population is fraught with instruction, and is suggestive of prudence, it is calculated to inspire a calmer confidence in the ordinances of nature, and to confirm our faith in the destinies of England. The expansion of which the reproductive force in the population is susceptible, and the progress of science and industry, must set at rest all dread of depopulation; which has apparently never prevailed for any length of time since the earliest historical ages. The population, it has been proved, has increased in a geometrical progression ever since the first census in 1801: and the rate of progression has been such that, if it continue, the numbers will have doubled in 1850: double the number of families will exist, and must be supplied with subsistence in England: but there will also be double the number of men to create subsistence and capital for her families, to man her fleets, to defend her inviolate hearths, to work the mines and manufactories, to extend the commerce, to open new regions of colonization; and double the number of minds to discover new truths, to confer the benefits and to enjoy the felicity of which human nature is susceptible." 141.

"The fallacy to which I have referred rests on this doctrine: 'the population is increasing in a geometrical progression, the means of subsistence in an arithmetical progression, and unless wars, destructive epidemics, marshes, dense towns, close workshops, and other deadly agents, carry off the excess of the numbers born—unless the outlets of life and blood be left open—the whole people must be exposed to a slow process of starvation.' This has been considered by some the doctrine of population. The nature of the increase in geometrical progression has been already examined; and there is no evidence whatever to prove that while capital increases in geometrical progression (compound interest) the subsistence and power of the people of these islands have increased, or will increase, in arithmetical, and not in geometrical progression. It is not known how much subsistence has increased in the last 40 years; and it is pure empiricism to pretend to say that the rate of progression has been, or will be arithmetical, if anything more be meant by that formula than the plain incontrovertible fact that the increase of subsistence is limited. But independently of these considerations, and any matters of controversy which it would be inconvenient to advert to here, the facts in the previous part of this paper dispose of the fallacy,—which, if it cannot be employed by any but the most depraved to sanction the destruction of life, might slacken the zeal of some in ameliorating the public health, by lending a colour to the dreadful notion that the excess of population is the cause of all the misery incidental to our condition or nature; and that the population might at the same time be diminished and saved from starvation, by epidemic diseases, unhealthy employments, or pestilential localities. What are the facts? An increase of the deaths can only diminish the population if the number of births remain stationary. It has been shown that the number of births may be increased to an incredible extent; experience has proved that the births almost invariably increase when the mortality increases; and it will be seen, in the Tables of the

Report, that where the mortality is greatest, the births are most numerous, and the population is increasing most rapidly. An increase of the mortality is therefore no specific for establishing an equilibrium between subsistence and population. The more, in fine, the doctrines of population are studied, the more deeply must be impressed upon the mind the sacredness of human life, and of the safeguards by which it has been surrounded by God and the laws." 143.

The Second Paper, devoted to the registration of the *causes of death*, is chiefly explanatory of the nosological arrangement adopted, furnishing, for the information of the Registrars of Districts and the profession generally, a statement of the chief objects to be kept in view in reference to the registration

It is prefaced by the invitation of the Presidents of the Colleges of Physicians and Surgeons and the Master of the Society of Apothecaries, circulated in May, 1837, to "all authorised practitioners throughout the country to follow their example, and give, in every instance which may fall under their care, an authentic name of the fatal disease, and to assist in establishing a better registration in future throughout England."

In reference to this subject some question has lately been raised as to the equity or fairness of calling upon members of the medical profession to contribute the information required by the Registrars, as adding one more to the already large demands made upon their gratuitous labour by Poor Law Guardians and public institutions—a question no doubt which never would have been raised were there not too many examples constantly before them of an ungenerous effort, first to undervalue, and secondly to profit by, professional time and services, left without remuneration.

So important however to mankind do we consider a complete and accurate registration of the causes of death, that we would fain hope—even though the legislature and government may have thrown upon medical men the onus and labour of providing the information at their own cost, —which by its value and importance might well have merited remuneration—that nevertheless it will not be withheld.

The explanatory statement thus describes the dependence of the Registrars upon the profession, and the public objects to be attained by their assistance.

"The recent Act for registering Births, Deaths, and Marriages in England, presents an opportunity for obtaining that great *desideratum* in medical statistics, a more exact statement of *the causes of death*, in the case of every registered death throughout the whole of England and Wales, after the month of June next ensuing.

"The Register-Books in which all deaths are to be registered after the last day of June, 1837, contain columns wherein may be inserted the *cause of death*, in juxtaposition with those other important illustrative circumstances, the *sex*, the *age*, and the *profession* or calling of the deceased person. Each Register-Book will also be assigned to a particular District of small extent, and will thus show in what part of the kingdom each death has occurred. If, therefore, the cause of death be correctly inserted, there will exist thenceforward public documents, from whence may be derived a more accurate knowledge, not only of the comparative prevalence of various mortal diseases, as regards the whole of England and Wales, but also of the *localities* in which they respectively prevail, and the *sex*, *age*, and *condition of life* which each principally affects.

"For the attainment of this object, it is necessary to ensure, as far as it is possible, the correct insertion of the '*cause of death*.' It is obvious that on this subject the requisite information can seldom be given to the Registrar, except by the medical attendant of the deceased person; and that even if the Registrar be a medical practitioner (which in many instances will be the case), yet will he often be unable to ascertain the truth in this respect, if he is to depend solely on the reports of persons ignorant of medicine and of the names and nature of diseases; and it cannot be expected that from his own knowledge he will be able so far to correct their errors as to ensure a statement worthy of credit. The requisite information must therefore be supplied either *directly* or *indirectly* by the medical attendant of the deceased person;—that is to say, if such medical attendant is not applied to by the Registrar, he must afford the requisite information to those other persons to whom the Registrar must apply." 145.

In the following observations we cordially agree, referring to the importance of uniform statistical nomenclature.

"The advantages of a uniform statistical nomenclature, however imperfect, are so obvious, that it is surprising no attention has been paid to its enforcement in bills of mortality. Each disease has in many instances been denoted by three or four terms, and each term has been applied to as many different diseases; vague, inconvenient names have been employed, or complications have been registered, instead of primary diseases. The nomenclature is of as much importance in this department of inquiry as weights and measures in the physical sciences, and should be settled without delay." 145.

The worthlessness of vague information can not be more strikingly exemplified than in statistical inquiries. We would gladly see Miss Edgeworth's maxim in letters of gold in every hospital and in every sick room. "He that would make his knowledge useful must first be at the pains to make it exact."

This want of exactitude in observing and recording offers in truth the greatest obstacle to the attainment of the highest results of a general registration. Mr. Farr remarks that many of the entries are incorrect—many not furnished by medical men, and in vague and ill defined terms, such as "*decline*—fit—inflammation—visceral disease—cold—long illness!" &c. So also local terms are employed which appear to denote different diseases in different parts of the country.

This suggested the necessity of introducing, as far as might be found practicable, the use of a uniform intelligible nomenclature, and under the head "statistical nosology" a nomenclature will be found obviously framed with great care and judgment, and we think it upon the whole tolerably complete and satisfactory, and even were it less so, the importance of adopting uniform nomenclature throughout the country in reference to these returns is so great, that we strongly urge upon the profession a strict adherence to this statistical nosology—nor will time be thrown away in a careful perusal of the notes appended to the various classes of disease, with references to the authors whose writings represent the prevailing medical opinions which in a great measure guide the English practitioner, and who are cited therefore, either because they have given summaries of the present state, or have extended the domain of our knowledge in those particular branches by original investigations. As the object has been to refer to writings easily accessible, the names of few foreign or ancient writers have been given, and it will be found that the writers at the end of each class have generally treated more especially of the diseases of the class.

At the conclusion of the nosology, a well-digested paper is given, headed "Analysis of Morbid Phenomena—Nomenclature," in which Mr. Farr passes rapidly in review the elementary phenomena of disease, and considers more particularly how—

"The numerous, and, in some instances, apparently arbitrary species have been distinguished by original and systematic writers; for without admitting the assertion repeated by Cullen, that 'species are created by nature, genera by the human mind,'—as our ideas both of species and genera are creations of external nature *and* of the percipient mind,—the determination of these primary elements of generalization is unquestionably more important than the subsequent steps in the process, because an error here will be irreparable. The species in the statistical nosology occur in the registers as well as in all the systematic medical works; and my object is not so much to propose anything new, either in the names or the species, (it being the very nature of an arrangement of the facts observed by all the practitioners of a country, to follow, as the observers themselves follow, the discoveries of pathology), as to point out some of the principles which have guided us in the distinction of species, and in the formation of the other divisions of the classification." 186.

The following observations give the key to the whole of the nosological arrangement advocated by the writer.

"In the constitution of species, more attention is now justly paid to structural than to functional changes; the former are often the proximate causes of the latter; but some pathologists, led astray by a principle of classification applicable to natural history,* or pre-occupied by their anatomical studies, and the recent discoveries in morbid anatomy, have denied the existence of dynamic disease; and, by a violent and improbable hypothesis, have assumed that every case, for instance, of insanity, convulsion, or syncope, is the *symptom* of a congestion, inflammation, or some other evident anatomical lesion. It would be as reasonable to assume that the needle of the mariner's compass never loses its magnetic properties but by evident oxidation.

"Upon an examination of the registers of the fatal diseases in the first years of registration, made, as is evident, from the instructions, without any preconceived notions on classification, it was found that, exclusive of epidemic diseases, a majority of the cases had been referred to particular organs, which were named or unequivocally indicated by the nature of the lesion. In other cases, such as hæmorrhage, dropsy, abscess, mortification, and cancer, the seat of the disease was seldom mentioned. The first class was arranged in groups, as sporadic diseases of the nervous, circulating, respiratory, digestive, urinary, generative, locomotive, and integumentary systems; the second, as diseases of uncertain seat

---

* "Pour que chaque être puisse *toujours se reconnaitre dans ce catalogue*, il faut qu'il porte son caractère avec lui: on ne peut donc prendre les caractères dans des propriétés ou dans des habitudes dont l'exercice soit momentanée mais ils doivent être tirés de la conformation: *Cuvier*—Règne Animal, tome i. p. 7. The problem in natural history is or *was*—Given one of many thousands or millions of individuals, what is its name and place in the 'catalogue?' As the specimen is often dead, or, as in fossils, has been only partially preserved, the superior importance of characters derived from the most permanent structures of the organization is obvious. Recognition is not a main object of any classification of diseases; and the most expert anatomist would, in numberless instances, find it impossible to divine from the after-death appearances the previous pathological phenomena."

(*de incertis sedibus.*) This mode of viewing the facts is common in England; it has been adopted in the treatises on the practice of physic which are most generally in the hands of practitioners; and, what is of more importance, by the authors who have devoted themselves successfully to research, and have naturally contributed most to the formation of the reigning medical opinions. The Library of Practical Medicine has followed this arrangement; and we have the original works of Abercrombie and Marshall Hall, on the Diseases of the Nervous System; Hope, on the Diseases of the Circulating System; Williams, on the Diseases of the Chest; Abercrombie, on Diseases of the Stomach and Intestines; Prout and Sir Benjamin Brodie, on the Diseases of the Urinary Organs; Willan and Bateman, on Cutaneous Diseases; not to mention others, and the treatises on midwifery, or the surgical treatises on the diseases of the joints and bones. Upon the other hand, there are essays and papers by Carswell, Watson, Sir James Clark, Mueller, Carmichael, and Walshe, on hæmorrhage, dropsy, tubercle, cancer, with a subordinate reference to the parts affected. The French writers, Laennec, Andral, Chomel, Rostan, Lallemand, and Louis, from whom we derived so much, have cast their practical works in the same mould. This mode of grouping and considering the different types of sporadic disease appears to be practically the best—to involve few errors in carrying it out, to lead to useful results, and to be in conformity with the general principles upon which diseases have been constituted and named.

"It will be observed that the different heads in the statistical nosology are numbered and sometimes subdivided; they may be called species, provided the term be not understood in the strict sense it bears in natural history:* with the technicalities of which medical science should not be encumbered, as it has principles of its own, and can derive more advantage from the methods of chemistry and natural philosophy." 190.

We have no hesitation in recommending this paper to the attention of all practitioners. It comprises observations on the causes and nature of diseases—a portion of which we have just quoted—on sporadic diseases of uncertain and variable seat—on inflammation—local diseases—diseases of the nervous system—of the circulatory, respiratory, and digestive organs —of the diseases of the urinary and locomotive organs—on diseases produced by poison—epidemic, endemic and contagious diseases—which he proposes should be called zymotic diseases for reasons given—viz.

"It must be admitted, with respect to all the forms of these diseases, that the body, in the cycle of external circumstances through which it passes, may run into them spontaneously (in this they differ from the class of diseases referred to external causes); for it is impossible to trace them invariably to infectious sources; it is not *à priori* more improbable that they than that other diseases should arise spontaneously; and it is impossible to account for their existence in the world upon any other principle than that of spontaneous origin. Still the property of communicating their action, and effecting analogous transformations in other bodies, is as important as it is characteristic in these diseases, which it

---

* "La génération étant le seul moyen de connaître les limites auxquelles les variétés peuvent s'étendre, on doit définir, *l'espèce la rèunion des individus descendus l'un de l'autre, ou de parents communs,* et de ceux qui leur ressemblent autant qu'ils se ressemblent entre aux.—*Cuvier,* R. A., tome i. p. 17. With this definition before our eyes, we cannot confound the species and genera of natural history with those of diseases."

is proposed therefore to call, in this sense, zymotic.* A single word, such as *Zymotics*, is required to replace in composition the long periphrasis 'epidemic, endemic, and contagious diseases,' with a new name, and a definition of the kind of pathological process which the name is intended to indicate, persons who have not made themselves acquainted with the researches of modern chemistry can scarcely fall into the gross error of considering this peculiar kind of diseased action and vinous fermentation absolutely identical, or of considering that others entertain that opinion. Liebig draws a distinction between fermentation and putrefaction; the reasons are more urgent for distinguishing the pathological transformations from fermentation or putrefaction, while it is admitted that they are of a chemical nature, and analogous to fermentation; by which they are moreover to a certain extent explained, although so little is known of the series of chemical changes and products in any single zymotic malady, or of the chemical re-actions of the living forces and organs. Small-pox is by hypothesis the transformation of varioline, and certain unknown concomitant chemical changes in the blood and skin; manifesting the important symptoms which fall under direct observation." 201.

On this subject, Mr. Farr has made many suggestions and reflections, if not altogether original, yet sufficiently novel in some of their applications to attract and repay attention.

It has been well said, by an eloquent writer, whom the world has lately lost, "who combines anew the knowledge received from other minds, explores its hidden and multiplied relations, and gives it forth in fresh and higher forms, is the rare possessor of an original mind." And, fully agreeing with Sir Philip Sidney, that "thinking nurseth thinking," we strongly recommend the paper as furnishing excellent materials for thought.

In describing the different effects of poisoning there is a pithy condensation of known facts and a suggestion for nomenclatures founded upon an hypothesis—that would undoubtedly be attended, in one sense, with many advantages.

"In very minute doses poisons have no visible effect, or have a sanative effect on the organization; in large doses their action is generally violent and local (acute); in moderate, long-continued, repeated doses, characteristic series of effects are produced, such as the mercurial salivation, erythema, and tremor, lead colic, and paralysis. If small doses of substances, poisonous in larger doses, are innocent or remedial, food in excess acts by its quantity like a poison, and gives rise to acute indigestion, or to the trains of symptoms indicated by gout, plethora, bloated obesity. The alimentary liquors, of which alcohol is the basis, intoxicate in large doses; and frequent intoxication induces delirium tremens, cirrhosis, and other pathological phenomena, which may be designated

* "From ζυμοω, I ferment: zymosis fermentation, and zyma ferment, may also be employed in English, *not in the sense* which they have in Greek, but as general designations of the morbid processes and their exciters. *Zymosis*, and the verb from which it is derived, occur in Hippocrates. See a good note and quotation from Galen, by Fœsius, in the *Œconomia Hippocratis*, appended to the Gêneva edition (1662) of the works of Hippocrates. *Coction* appears to have been used by the father of medicine with the same qualification as ebullition and fermentation by Sydenham. See his Treatise on Ancient Medicine, vol. i., Œuvres complètes d'Hippocrate, par E. Littreé, 1839."

*alcoholia*. The privation of the various kinds of fluid and food every day required by the organization, gives rise to acute symptoms (such as occurred, for instance, in the shipwreck of the Medusa), or chronic forms of disease, such as scurvy, and the malady that decimated the Millbank Penitentiary; the body, acted on by oxygen, when it can no longer serve for food, becoming a poison to itself, or falling spontaneously into states of disorganization analogous to the effects of poisoning. The special disease arising from privation might be called *pinia* (πεινα, famine, hunger); from high living, *obsoponia*, (οψοπονος, 'elaborately cooking victuals'); from cold, *psychria* (ψυχρος, cold); from heat, *thermia* (Θερμη, heat); without discarding the common terms starvation, scurvy, over-heating, gout, chilblains, frost-bitten (cold?), gangrene, coup de soleil, and some tropical diseases, or replacing the names of diseases which owe their origin indirectly to the excess or deficiency of food and warmth.

"Certain matters which have not yet been analysed produce small-pox, glanders, hydrophobia, syphilis, measles, scarlatina, and other diseases; and as it was before proposed to give names to the well-defined diseases produced by poisons, so, for the purposes of reasoning, it will be equally useful to name these specific matters or transformations of matter by which diseases are propagated either by inoculation and contact (contagion), or by inhalation (infection). The following list exhibits the popular and scientific names of diseases in juxta-position with the proposed names of their exciters; and it may be assumed hypothetically, that in the blood corresponding bodies exist, which are destroyed, and by the transformation of which the exciters are generated or reproduced. The names in the second column terminate in *a*, except a few in *s*. Lyssa (from λυσσα, rabies), the old Greek term, has been restored by Mason Good; I propose, for the sake of uniformity, to call puerperal fever, *metria;* mumps, *parotia:* reserving parotitis for simple inflammation of the parotids; croup, *trachea:* and the disease from puncture in dissection, *necusia*, (νεκυς, the dead body.)

| *Diseases.* | | *Zymotic Principles.* |
|---|---|---|
| Small-pox .. .. .. | variola | varioline. |
| Cow-pox .. .. .. | vaccinia. | vaccinine. |
| Glanders.. .. .. .. | equinia. | equinine. |
| Hydrophobia.. .. .. | lyssa. | lyssine. |
| Syphilis .. .. .. .. | syphil*is*. | syphiline. |
| Infection in dissecting .. | necusia. | necusine. |
| Erysipelas .. .. .. | erysipel*as*. | erysipeline. |
| Puerperal fever .. .. | metria. | metrine. |
| Measles .. .. .. .. | rubeola. | rubeoline. |
| Scarlet fever .. .. .. | scarlatina. | scarlatinine. |
| Hooping-cough .. .. | pertuss*is*. | pertussine. |
| Dysentery .. .. .. | dysenteria. | enterine. |
| Diarrhœa .. .. .. | diarrhœa. | |
| Cholera .. .. .. .. | cholera. | cholerine. |
| Influenza .. .. .. | influenza. | influenzine. |
| Typhus .. .. .. .. | typh*us*. | typhine. |
| Plague .. .. .. .. | pest*is*. | pestine. |

"The existence of gangrenine, ergotine, ophthalmine, tetanine, miliarine, diphtherine, parotine, aphthine, tracheine, may also be admitted. It is maintained by some pathologists, that the same specific poison produces several of these diseases—erysipelas, necusia, and metria, for instance; but while the diseases are described as distinct, it will be most convenient to consider their exciters as distinct, although they may be convertible into each other, and be as nearly related as varioline and vaccinine." 200.

Into the theory of hypothesis on which the production of zymotic diseases is attempted to be explained, the writer enters at some length, resting his conclusions mainly upon the older writers, when these diseases were so rife in London, as to divert attention from pure inflammations, and he observes—

"The early medical observers have directed attention to the analogies zymotic diseases have with combustion, fermentation, putrefaction, and poisoning. These analogies have been, to a certain extent, confirmed by the researches of modern chemistry; and Liebig has been led by the study of organic transformations—fermentation, putrefaction, decay—to develope a theory invented by the greatest practical physicians to explain the phenomena of zymotic diseases.

"Liebig observes, 'that physicians had referred formerly to fermentation merely by way of illustration;' from which it is evident that he had not had time to consult the English medical classics on this head, or he would have discovered, not, indeed, an anticipation of his own admirable generalizations, but a theory very similar to his own—the basis of their pathology—founded upon enlarged views, and well calculated to prepare the way for his researches and the researches of other chemists." 204.

And again—

"The three great contemporaries, Sydenham, Morton, and Willis, lived in London when plague and epidemic diseases prevailed; and much as they differed, or were mistaken on some points, all announced more or less clearly the zymotic hypothesis. They were not, it must be borne in mind, mere chemiatric theorists; they had studied diseased action as assiduously, and with as much sagacity, as modern chemists have studied fermentation; Willis was a great anatomist; Sydenham and Morton have left original pathological delineations, which have never been surpassed, and laid down plans of treatment which are still followed.

"Liebig, Dumas, and the chemists of this country, will, we sanguinely hope, not rest satisfied with what has been done, but continue to prosecute their labours with ardour and success; and, from the study of the series of transformations of nitrogenous compounds, proceed to investigate the transformations of the blood, tissues, and secretions which accompany the production of varioline, typhine, and the other zymotic principles." 205.

This part of the volume concludes with a note in reply to some observations on the "Report of a Sub-Committee of the Royal College of Physicians in Edinburgh." Mr. Farr very justly remarks.

"There is nothing perhaps in which it would be more difficult to get all medical men to agree than in any one classification of diseases; and though this is partly due to the imperfect state, it cannot be entirely ascribed to the uncertainty, of medical science, for it really arises from the utility, not to say the necessity, of considering the facts in different combinations, according to the object in view. The nosology which was given in the first Report, and of which the present is an extension, has been received quite as favourably as was anticipated: it has been employed, with some modifications, for statistical purposes in this country and abroad. In the present revision I have endeavoured to profit by the friendly criticism of the medical press, and the suggestions with which I have been favoured by members of the profession, who naturally take an interest in a national system of registration." 206.

Into the critique however or the reply we deem it unnecessary to enter, nor indeed will our space permit further notice than to observe that, we think Mr. Farr rightly says he has to thank Dr. Allison and his colleagues for affording him an opportunity of "giving some explanations and answering some objections against the statistical nosology, which could not perhaps have otherwise been conveniently given or answered here." From this explanation we extract the following statement of the principle of registration adopted.

"The plan in England has been to recommend medical practitioners to return the causes of death under specific names; to distinguish, where they can, all the diseases which are recognised as distinct in the present state of science; to return in general terms the diseases which are imperfectly known, and not to assign any cause without satisfactory evidence of the accuracy of their information. To promote uniformity in the nomenclature, a list of the diseases which are considered distinct in the standard medical works of the day, has been drawn up, and *recommended* for adoption, if the medical informant should consider that the names there given express the cause of death with sufficient accuracy. In the present stage of registration, however, these causes are referred, in the published abstracts, to a much smaller number of heads; comprising the diseases of frequent occurrence, or diseases which it appears interesting, under one point of view or other, to consider apart." 215.

An abstract is given with the tabular returns—showing the state of the public health in 1840. We confess we are at a loss to know why a Report of the Registrar-General published late in 1842 should only furnish an abstract of the public health for the year 1840—or why an analysis and statement is not furnished for 1841, more especially as this volume includes tables of mortality to the 21st of May, 1842, and Major Graham in his report, dated August 8th, 1842, states that,

"To ascertain the state of the public health down to the latest time, I have directed to be prepared a quarterly table of the mortality in 114 of the principal districts (including nearly all the large towns) of England; showing the average quarterly deaths, and the number of deaths registered in the quarter immediately preceding the publication." 19.

Showing that the latest intelligence was considered desirable.

Questions of public health are among the most important that can engage the attention of the medical philosopher—nor is their interest in any sense confined to these limits—inquiries into the causes, physical, political and moral, influencing and determining the public health, are worthy of general attention and study—nay, interest all—the poor by their physical well-being—rate-payers by their pockets—governments by policy—and the enlightened by philanthropy.

The abstract before us shows that there is much yet to be done before a complete registration can be attained. In the year 1840, the supposed causes of 351,757 deaths were stated, leaving 7,804 deaths in which the causes were not stated.

"The mortality by all causes was higher in 1840 than in 1838, and considerably higher than in 1839. Out of 1,000,000 living in 1839, only 21,856 died; while in 1840, out of the same number living, 21,856—and 1,022 more perished. Upon referring to the deaths by different classes of causes, it will be perceived that 626 of the excess (1,022) arose in the epidemic class of diseases; the remaining excess being distributed over all the classes, except that of the violent deaths, which diminished somewhat in each of the two last years.

"The deaths from *Small-pox* fell from 16,268 in 1838, to 9,131 in 1839, and 10,434 in 1840; the deaths from *Typhus* fell from 18,775 to 15,666 and 17,177.

From both diseases the mortality was less in 1840 than in 1838, but greater than in 1839. *Hooping-cough* progressively declined from 9,107 deaths, to 8,165 and to 6,132." 218.

Scarlatina, it appears, was the reigning epidemic of the year 1840. The deaths from it in the three years were—

| 1838, | 1839, | 1841, |
|---|---|---|
| 5,802. | 10,325. | 19,816. |

The epidemic was most destructive in the North-western, North-midland, York, Welsh, and Northern Divisions. The epidemic had not terminated at the close of the statistical year.

" The deaths by *Diarrhœa, Cholera, Influenza,* and *Ague,* increased to a considerable extent; though not so as to assume the epidemic form, or to present anything very remarkable.

" The deaths from *Hydrophobia* in the three years were 24, 15, 12; and therefore not half so numerous in 1840 as in 1838.

" The mortality by the diseases of the *Nervous System* was nearly the same in the three years, *viz.,* ·003365, and ·003255, and ·003302. The mortality by *Cephalitis* and *Paralysis* was slightly higher in 1840 than in the two preceding years.

" The mortality by the diseases of the *Respiratory Organs* was 6 in 1,000; or in each of the three years ·006149, ·005989, and ·006043. The deaths ascribed to *Consumption* in each of the three years were 59,025, 59,559, and 59,923; and the mortality was ·003996, ·003939, and ·003897. The mortality from this disease declined very slightly. About 4 in 1000 persons died annually of Consumption, and about one-fifth or one-sixth part of the total deaths was by this disease.

" The mortality of diseases of the *Digestive Organs* was ·001307, ·001373, and ·001465. The increase was chiefly in *Enteritis.*

" The mortality by diseases of the *Urinary Organs* was ·000112, ·000101, and ·000110, in the three years. The deaths by *Stone* (and *Gravel*) were 320, 299, and 303: the mortality ·000022, ·000020, and ·000020. About 1 in 50,000 persons die of stone annually. It will be interesting to see whether the mortality be reduced in future years by the discoveries of surgery. The mortality by *Diabetes* is to that by *Stone* as nearly two to three.

" The deaths in *Childbed* were 2,811, 2,915, and 2,989 in the three years. The mortality increased from ·000190 to ·000193, and ·000195. To about 187 children born alive, *one mother died.* The proportion of mothers who perish at this important period is unquestionably excessive; and must suggest to every humane person the inquiry whether the education of the nurses who attend the poor in labour may not be improved.?

" The number of deaths ascribed to *Rheumatism* and to diseases of the joints was 962, and 1,170 in 1840.

" If we except ' Debility,' under which head are included, ' premature births,' —' Dropsy' was the most fatal of the diseases of ' *uncertain or variable seat.*' The deaths ascribed to dropsy were 12,342, 12,251, and 13,261 in the three years; the annual rate of mortality ·000836, ·000810, and ·000863. It is scarcely necessary to add, that according to the present views of pathologists, *Heart Disease,* or *Nephria,* would in the majority of cases be considered the primary affections. So difficult, nevertheless, appears to be the diagnosis practically, that nearly as many cases of simple ' Dropsy' are registered in the London Hospitals as out of doors in private practice." 218.

An interesting table is given of the relative mortality by different causes in the three years 1838-40. The greatest number occur in the following classes.

| | |
|---|---|
| Diseases of respiratory organs .. .. .. | 92,907 |
| Epidemic, endemic, and contagious diseases, | 76,064 |
| Nervous system .. .. .. .. .. .. .. | 50,768 |
| Organs of Digestion .. .. .. .. .. .. | 22,325 |
| | 242,264. |

There are three other large classes in which the information is more vague and probably less to be depended upon, viz.

| | |
|---|---|
| Diseases of uncertain seat .. .. .. .. .. | 48,396 |
| Old age .. .. .. .. .. .. .. .. .. .. | 36,793 |
| Deaths ascribed to external causes, cold, violence | 11,922 |
| | 97,111 |
| Deaths above specified in four classes .. .. | 242,264 |
| | 339,375 |

Total number of deaths returned by all causes is.. 359,561 } These two divisions therefore leaves

339,375 }

only.. ·20,186 to be accounted for by the remaining six classes enumerated.

And of this 20,000, the causes are not ascertained in nearly 8,000.

We take our leave of this volume, which we may safely affirm, from its very moderate bulk, contains more valuable information, and affords more abundant materials for thought and enquiry of the most useful kind, than a very large proportion of the books which issue each season from the press could yield, were they all put together, and winnowed of their chaff. With this opinion we need not say that we strongly recommend it to the study of the profession generally, whom it behoves more especially to be well informed, on all subjects touching the public health, and heartily do we concur in the hope expressed by the Registrar-General at the conclusion of his Report.

"I hope that the registrars and informants will not fail to see the necessity of attending to the classification of fatal diseases, which has been framed for their use with much labour and after careful consideration; and I also trust that the members of the medical profession, who have hitherto given their aid, will cordially assist in carrying out this national registration of the causes of death; as they alone are enabled to give a correct statement of the nature of the fatal diseases; and to them, more than to the members of any other profession, must be apparent the vast importance of thus collecting accurate materials for advancing the science of vital statistics." 24.

